# Radiation impacts gene redundancy and biofilm regulation of cryoconite microbiomes in Northern Hemisphere glaciers

**DOI:** 10.1186/s40168-023-01621-y

**Published:** 2023-10-18

**Authors:** Zhihao Zhang, Yongqin Liu, Weishu Zhao, Mukan Ji

**Affiliations:** 1 State Key Laboratory of Tibetan Plateau Earth System, Resources and Environment (TPESRE), Institute of Tibetan Plateau Research, Chinese Academy of Sciences, Beijing, 100101 China; 2 University of Chinese Academy of Sciences, Beijing, 100049 China; 3 Center for Pan-Third Pole Environment, Lanzhou University, Lanzhou, 730000 China; 4 State Key Laboratory of Microbial Metabolism, School of Life Sciences and Biotechnology, Shanghai Jiao Tong University, Shanghai, 200240 China; 5 SJTU Yazhou Bay Institute of Deepsea Sci-Tech, Yongyou Industrial Park, Sanya, 572024 China; 6 International Center for Deep Life Investigation (IC-DLI), Shanghai Jiao Tong University, Shanghai, 200240 China

**Keywords:** Cryoconite microbiome, Metagenomic, Radiation, Biofilm, Niche, Glacier ecosystem

## Abstract

**Background:**

Glaciers harbor diverse microorganisms adapted to extreme conditions with high radiation, fluctuating temperature, and low nutrient availability. In glacial ecosystems, cryoconite granules are hotspots of microbial metabolic activity and could influences the biogeochemical cycle on glacier surface. Climate change could influence glacier dynamics by changing regional meteorological factors (e.g., radiation, precipitation, temperature, wind, and evaporation). Moreover, meteorological factors not only influence glacier dynamics but also directly or indirectly influence cryoconite microbiomes. However, the relationship of the meteorological factors and cryoconite microbiome are poorly understood.

**Results:**

Here, we collected 88 metagenomes from 26 glaciers distributed in the Northern Hemisphere with corresponding public meteorological data to reveal the relationship between meteorological factors and variation of cryoconite microbiome. Our results showed significant differences in taxonomic and genomic characteristics between cryoconite generalists and specialists. Additionally, we found that the biogeography of both generalists and specialists was influenced by solar radiation. Specialists with smaller genome size and lower gene redundancy were more abundant under high radiation stress, implying that streamlined genomes are more adapted to high radiation conditions. Network analysis revealed that biofilm regulation is a ubiquitous function in response to radiation stress, and hub genes were associated with the formation and dispersion of biofilms.

**Conclusion:**

These findings enhance our understanding of glacier cryoconite microbiome variation on a hemispheric scale and indicate the response mechanisms to radiation stress, which will support forecasts of the ecological consequences of future climate change.

Video Abstract

**Supplementary Information:**

The online version contains supplementary material available at 10.1186/s40168-023-01621-y.

## Background

Glaciers and ice sheets cover approximately 10% of the global surface and these icy ecosystems are dominated by microbes, which drive biogeochemical cycling [1]. The Northern Hemisphere contains over half the glaciers on Earth, and the mass balance of these glaciers is greatly influenced by climate change. Climate change could influence glacier dynamic by changing regional meteorological factors (e.g., solar radiation, temperature, precipitation, wind, and evaporation). For instance, divergent summer insolation has been shown to be the major driver of long-term glacier evolution [2]. In addition, the microbial community on the glacier surface is directly or indirectly influenced by meteorological factors. Among meteorological factors, solar radiation is fundamental in glacial ecosystems because it supports phototrophic microorganisms and serves as a permanent driving force in glacier evolution. Recently, it has been reported that solar radiation (light) stimulates primary production in cryoconite and directly supplements the energy demand of aerobic anoxygenic phototrophs [3, 4]. Moreover, microbial processes at the surface have the potential to amplify the impacts of meteorological factors on glaciers [5]. Therefore, understanding how microbial communities respond to meteorological factors is the key to making reasonable predictions of the microbiome and glacier dynamics, contributing to estimating the impacts of climate change.

Cryoconite is a granular sediment found on glacier surfaces comprising both mineral and biological materials from different sources, recognized as unique hot spots of microbial diversity and activity in glacial ecosystems [6]. The pioneers in cryoconite are cyanobacteria, which produce substantial amounts of organic material, including extracellular polymeric substances (EPS), which hold minerals and other particles together, effectively increasing the lifetime of cryoconite on the ice surface [7, 8]. Moreover, other microbes in cryoconite can also produce EPS that allow them to form biofilms and increase their survivability, such as cryoprotection, anti-desiccation, buffering against high salinity and pH, and trace metal uptake and binding [9–12]. Smith et al. (2016) revealed that ~ 35% of the cryoconite sediment surfaces were covered by biofilm in Antarctica [13]. The accumulation of microbial biofilms will enlarge these aggregates, potentially contributing to surface darkening and the reduction in glacial albedo [6]. Given that light could increase the excretion of cyanobacterial EPS [14], we propose the presence of a linkage between the biofilm of the cryoconite microbiome and solar radiation.

In recent years, many studies have reported that cryoconite ecosystems are usually dominated by endemic species owing to geographic separation [15, 16], e.g., Millar et al. (2021) revealed that specialists dominated the cryoconite community on the hemispheric scale [17]. In general, the overwhelming majority of microbial ecosystems are characterized by highly skewed abundance-rank distributions: a few taxa account for the majority, while most taxa are represented by only a few individuals [18]. According to the difference in taxon distributions among habitats, taxa with equal abundances in many habitats are divided into generalists, whereas taxa that are always found in only one habitat are divided into specialists [19]. Specialists are usually highly adapted to specific conditions and are sensitive to environmental change, whereas generalists are recognized as adapting to a wide range of environments [20]. Moreover, generalist and specialist microbes differently impact the dynamics of microbial community structures [21]. Hence, distinguishing the roles of generalists and specialists, including their habitat range and metabolic potential, is meaningful to understanding the dynamics of cryoconite ecosystems.

Overall, understanding the relationship between meteorological factors and dynamic of cryoconite microbiome requires an in-depth analysis of the roles played by different species as well as their habitat ranges and metabolic potential. Considering these factors, we seek to differentiate the roles of generalists and specialists, find their connection with meteorological factors, and indicate the potential mechanisms. In this study, we collected 88 metagenomes of the cryoconite microbiome from 26 sampling sites across glaciers in the Northern Hemisphere with corresponding meteorological data. Finally, we combined genome and gene-centric approaches that identify key species and functions of the community, as well as their connection with meteorological factors, to provide insights into the variation of cryoconite microbiomes under climate change.

## Materials and methods

### Metagenomic and meteorological data collection

We analyzed 88 metagenomes of the cryoconite microbiome from 26 glacier sampling sites distributed in the Northern Hemisphere with an average coverage of 1.27 × 10^10^ (± 9.76 × 10^9^) bases per metagenome (Additional file 1: Table S1). Owing to the sequence length of paired reads was below 100 bp, the metagenomic data of Rotmoosferner glacier was not include in this study [22]. All 88 samples were distributed among Northern Hemisphere glaciers and divided into three groups (i.e., ALP, ARC, and TP) based on their geographic position, including Alp (6 samples in Italy), Arctic (39 samples in Greenland, 3 samples in Svalbard, and 2 samples in Alaska) and Tibetan plateau as well as its surroundings (25 samples in China, 6 samples in Pakistan, 5 samples in Nepal, one sample in Tajikistan and one in Kyrgyzstan) (Additional file 2: Fig. S1). The 88 metagenomes of the cryoconite microbiome cover a broad range of temporal conditions from July to November from 2005 to 2020. Samples were taken, and DNA extraction from the cryoconite was performed as described in the original research [3, 23–26].

In addition, we downloaded monthly averaged meteorological data of 26 sampling points from ERA5 dataset, which is a global atmospheric reanalysis product produced by the ECMWF (European Centre for Medium–Range Weather Forecasts) [27]. Then we selected mainly meteorological data that could influence glacier as well as cryoconite microbiomes, including 2 m temperature (Kelvins, K), 10 m wind speed (meters per second, m/s), total sky direct solar radiation at the surface (joules per square meter, J/${m}^{2}$), total precipitation ( meter of water equivalent), and evaporation (meter of water equivalent) data of each sampling site from 2005 to 2020. The NetCDF (Network Common Data Form) format dataset was download in the website [28] and transformed to table format based on geographic coordinate by R package *raster* (v3.5–15) [29]. Finally, these meteorological data were converted to a 15-year average value for each sampling point for downstream analysis.

### Metagenome analysis

Raw reads of metagenomes were obtained from the National Center of Biotechnology Information (NCBI) Sequence Read Archive (SRA). Quality control of pair-end reads was performed by Trim Galore wrapper (v0.6.6) [30]. Megahit (v1.1.3) [31] was used to assemble high-quality reads with the default setting, and only contigs > 1000 bp were retained. A total of 32,787,657 assembled contigs were used to predict open reading frames (ORFs) by Prodigal (v2.6.3) [32], and then ORFs were clustered by MMseqs2 (v13.45111) [33] with the parameters: easy-linclust -e 0.001 -min-seq-id 0.95 and -c 0.80, as used in the human gut microbiome dataset construction except decrease 90% coverage to 80% for excluding the effect of shorter genes [34]. A total of 17,008,994 nonredundant ORFs were generated, with an average length of 665 bp. These ORFs were annotated against Kyoto Encyclopaedia of Genes and Genomes Orthology (KEGG) [35] databases using KofamScan [36] at an e value threshold of 1e − 5. Clean reads were mapped to nonredundant ORFs by Salmon (v0.13.1) [37] with default parameters to obtain transcripts per million (TPM) abundance.

### Genome binning and recruitment

Binning of the remaining assembly contigs and high-quality reads was performed by the variational autoencoders for metagenomic binning (VAMB) (v2.0.1) [38] and metaWRAP (v1.3.2) [39] with self-implemented MetaBAT2 [40] and MaxBin2 [41] binning modules. A total of 11,813 bins were generated by three binning methods. Then, all these sets of bins were pooled together for bin dereplication and aggregation by DAS_Tools (v1.1.4) with default settings [42]. A total of 2556 bins (also called metagenome assembly genomes, MAGs) were further refined to remove heterogeneous contig potential contamination based on the genomic properties (i.e., tetranucleotide signatures, coverage, and GC content) by RefineM (v0.0.24) [43]. The completeness and contamination of each bin were assessed with CheckM (v.1.0.11) using the lineage_wf workflow [44].

The 2078 medium–high quality MAGs with completeness > 50% and contamination < 10% were clustered at the species level by dRep (v3.2.2) [45] with an average nucleotide identity (ANI) threshold of 95% [25, 46] and an aligned fraction threshold of 30%. The final 645 dereplicated MAGs represented unique species that we called species-level genome bins (SGBs) as used in the human gut microbiome research [47] (Additional file 1: Table S2**)**. Taxonomic classification of SGBs was performed by GTDB-tk (v0.3.2) [48, 49] with the Genome Taxonomy Database (GTDB) (R06-RS202). SGB abundance was calculated by CoverM (v0.6.0) using the TPM method [50]. Protein sequences of SGBs were predicted with Prokka (v1.14.6) [51], and functional annotation was performed with the same pipeline used for ORFs. A phylogenetic tree for the dereplicated MAGs was reconstructed using the protein sequences of 43 universal single-copy genes by checkM. The iTOL website [52] was used to better visualize the phylogenetic tree.

### Niche breadth analysis

Specialist-generalist classification of SGBs was based on Levins’ niche breadth index. To avoid sampling bias, the function spec.gen from the R package *EcolUtils* (v.0.1) [53] was used to calculate Levins’ index for 1000 random permutations of the metagenomic TPM table. Then, 645 SGBs were categorized as generalists if Levins’ index was larger than its 95% confidence interval (CI) or specialists if Levins’ index was smaller than its 95% CI, and the SGBs were considered uncategorized if Levins’ index was within the 95% CI.

### WGCNA network analysis

A weighted gene co-expression network analysis (WGCNA) was performed using the R package *WGCNA* (v1.70–3) [54]. The analysis was performed on 88 samples of the KEGG dataset to describe networks derived from their normalized TPM values for information on gene and function abundances.

A total of 17,347 KEGG orthologs (KOs) were detected across 88 samples and then filtered to 8272 KOs that were observed in at least 70% of samples. Filtered KOs were transformed into centered log ratios (CLR) after replacement of zeros via count zero multiplicative replacement by R package *zCompositions* (v1.4.0–1) and *compositions* (v2.0–4) [55]. Then transformed data was used to construct the network based on the absolute values of Pearson correlation coefficients. To generate an approximate scale-free topology network, soft thresholding power was set at 7 by the function *pickSoftThreshold*. Network module identification was performed by a dynamic tree cut algorithm and the minimum module size was set at 50 to generate medium to large modules. Highly similar (> 75%) network modules were merged by hierarchical clustering, which resulted in 10 network modules being retained. The KOs within same network module had high correlation that could perform similar or relevant function; hence, we also called these network modules as function modules. The module’s eigengene values, which are equivalent to the first principal component, were examined for correlations with radiation using a Pearson correlation.

To evaluate the importance of modules to radiation, linear regressions were used to fit each module’s eigengene value and meteorological data. Before fitting, L1-regularized regression with the least absolute shrinkage and selection operator (LASSO) [56] was used to select the variables and avoid overfitting. For LASSO, the final selected variables were depended on the choice of lambda value. Under the best lambda, final three module (MEturquoise, MEblue, and MEgreenyellow) was selected using cross validation. Then, the eigengene value of selected modules was used to construct an optimization model. The importance of modules to radiation was evaluated by the standardized regression coefficient of the final model. The resulting regression estimates were visualized as forest plots with 95% confidence intervals by R package *forestmodel* (v0.6.2) [57]. All regression methods were performed using the R package *glmnet* (v4.1–4) [58].

Each module significantly correlated with meteorological factors was regarded as an environmentally responsive functional group in the cryoconite microbiome. To determine the represented function of each module, KEGG pathway enrichment analysis was performed using the R package *clusterProfiler* (v3.18.1) [59]. To find radiation-related hub genes in module MEturquoise and MEblue, KOs were filtered based on threshold: module membership over 0.9 and absolute value of radiation correlation over 0.8. Module membership value and radiation correlation value that measured by *signedKME* and *cor* function of *WGCNA* R package. Then, we check the pathway of filtered KOs and select the pathway also identified in enrichment analysis. The selected pathways were bacterial motility proteins, biofilm formation, flagellar assembly, O-antigen nucleotide sugar biosynthesis, quorum sensing, secretion system, and two-component system. Finally, the KOs of selected pathway were visualized in cooccurrence networks by the open-source tool Cytoscape (v3.7.0) [60].

### Statistical analysis

To verify the plausibility of biogeographical categories (i.e., ALP, ARC, and TP), clustering analysis was performed by *factoextra* (v1.0.7) and *cluster* (v2.1.3) R package. The CLR-transformed SGB abundances was explained by the meteorological factors using Redundancy analysis (RDA) by the vegan (v2.6–2) package in R. [61]. To assess collinearity between meteorological factors, the variance inflation factor analysis (VIF) was performed to filter redundancy variable with the *vif.cca* function. All meteorological factors with low variance inflation factor (VIF < 10) were kept. Then, we used the *rdacca.hp* (v1.0–8) package to distinguish the contribution of each meteorological factor by the relative importance of each factor independently accounting for variation in community structure [62]. In addition, to determine the relative importance of meteorological factors in structuring cryoconite communities, we also conducted a multiple regression analysis using the multiple regression on matrices (MRM) approach in the *ecodist* R package (v2.0.7) [63]. Linear regression between sum of KOs and unique KOs of each SGB was plotted using the stat_poly_eq function in the *ggpmisc* (v0.4.6) package in R, and then *p* values and *R*^2^ values were calculated and added to the graph [64]. The function *diffslope* in the R package *simba* (v0.3–5) was used to calculate the difference in slopes of regression lines. The correlation between 645 SGBs abundance (CLR transformed) and radiation was canulated by Spearman correlation test by rcorr function of R package *correlation* (v0.8.1). The significance of the SGB-based comparisons of predicted GC content, genome size, gene redundancy index, and radiation correlation among niche breadth groups was determined using the Wilcox rank sum test in the *ggpubr* (0.4.0) package in R [65]. All statistical analyses were performed using R version 4.0.3.

## Result

### Taxonomic composition of the cryoconite microbiome in the Northern Hemisphere

**Fig. 1:**
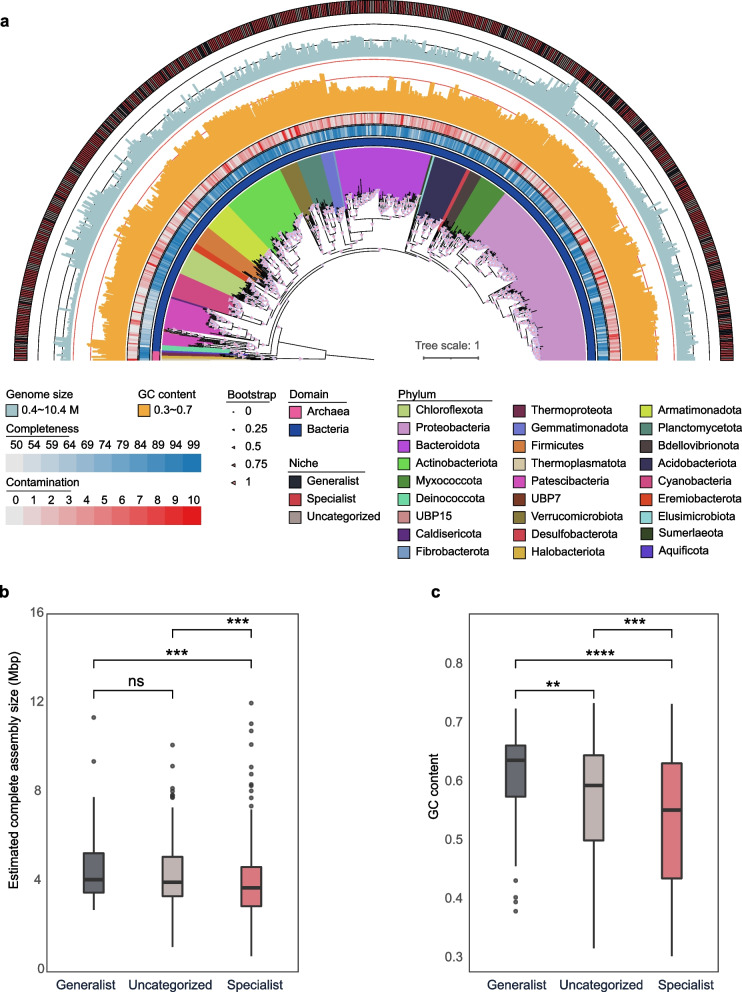
Phylogenetic tree of SGBs and genome characteristics of cryoconite microbiome. **a** Phylogenetic tree was generated using 645 SGBs. Bar plot shows the completeness (blue) and contamination (red) of SGBs. Color strip in the outer ring represents the niche breadth groups divided by Levin’s niche breadth, black for generalist, red for specialist, and gray for uncategorized. Bootstraps are shown in the pink triangle and are based on 1000 replicated trees. **b**, **c** Comparison of estimated genomes size and GC content between different niche breadth groups (Wilcox rank sum test). Significance level: **** (*p* ≤ 0.0001); *** (*p* ≤ 0.001); ** (*p* ≤ 0.01); * (*p* < 0.05)

After quality filtering and dereplication, 2556 MAGs were generated with an average mapping rate of 63.5% against 8,697,020,884 clean reads. To uncover the diversity of cryoconite species, 645 SGBs were obtained from 2078 medium–high quality MAGs by clustering at the species level based on an ANI threshold of 95% [25, 46]. These SGBs were affiliated with 27 phyla; 24 were bacteria, and the other three were archaea (i.e., Halobacteriota, Thermoplasmatota, and Thermoproteota) (Fig. 1a). All eight archaeal SGBs were classified at the family level. Four SGBs belonged to sulfur-oxidizing archaea in the families Sulfolobaceae and Thermoproteaceae. Three SGBs belonged to methanogenic archaea in the families Methanosphaerulaceae and Methanosarcinaceae and UBA472 in the order Methanomassiliicoccales. One SGB was classified to the ARK-15 family, which shared a common ancestor with the sulfur-reducing archaeon Aciduliprofundum [66]. At the genus level, six SGBs were classified, while at the species level, only two SGBs were classified as *Metallosphaera sedula* and *Methanosarcina sp001714685*. The other 637 bacterial SGBs were most frequently assigned to the phylum Proteobacteria (28.8%), followed by Bacteroidota (14.7%) and Actinobacteriota (9.6%). At the family level, 94% of bacterial SGBs could be annotated, and the most abundant family was Burkholderiaceae (8.3%), followed by Sphingomonadaceae (6.4%) and Chitinophagaceae (5.3%). At the genus level, 65% of bacterial SGBs could be annotated, and the most abundant genus was Ferruginibacter (5.5%), followed by Rhizobacter (3.4%) and Polaromonas (2.8%). However, only eight bacterial SGBs could be classified at the species level (Additional file 1: Table S2).

### Generalists and specialists in the cryoconite microbiome

We further divided 645 SGBs into two groups, generalist, and specialist, based on Levin’s niche breadth index [67]. In this study, only 12% of SGBs were identified as generalists, 55% of SGBs were specialists, and 33% of SGBs were uncategorized. The comparison of taxonomic composition between niche breadth groups showed that specialists exhibited higher diversity than generalists. Specialists were classified into 25 phyla and generalists into 13 phyla in this study. SGBs belonging to the phyla Proteobacteria, Bacteroidota, and Actinobacteriota were abundant in both the generalist and specialist groups. However, the number of the superphylum Patescibacteria (also known as candidate phyla radiation, CPR) was different between generalists and specialists, with only one in the generalist group and 30 in the specialist group. At the genus level, the SGBs in the generalist and specialist groups belonged to 34 and 125 genera, respectively. SGBs belonging to the genera Ferruginibacter, Polaromonas, and BOG-908 were abundant among the generalists and specialists. Sixteen genera were found only among generalists, and 107 genera were found only among specialists. In addition, uncategorized SGBs also showed highly diverse taxonomic composition. A total of 216 uncategorized SGBs were distributed among 20 phyla, most of which belonged to Proteobacteria (27%), followed by Bacteroidota (14%) and Actinobacteriota (10%). The phylum UBA15 was found only in an uncategorized group. At the genus level, SGBs were classified into 86 genera, and the genera Rhizobacter, Ferruginibacter and UKL13-2 were abundant. Additionally, there was no generalist archaeal SGBs. Five archaeal SGBs were specialist group and belong to three phyla, that is Halobacteriota, Thermoplasmatota, and Thermoproteota. While other three archaeal SGBs were uncategorized group and belong to Thermoproteota and Thermoplasmatota, two classified archaeal species (i.e., *Metallosphaera sedula* and *Methanosarcina sp001714685*) were specialist group.

Generalists and specialists exhibited obviously different genomic features. The average estimated genome size for generalists was significantly larger than that for specialists (Wilcox rank sum test; *p* value < 0.001), which were 4.58 (± 1.56) and 4.20 (± 1.50) Mbp, respectively (Fig. 1b). Similarly, the average GC of generalists (0.61 ± 0.08) was significantly higher than that of specialists (0.53 ± 0.11) (Wilcox rank sum test; *p* value < 0.0001) (Fig. 1c). For uncategorized SGBs, the average estimated genome size and GC content were intermediate to those of generalists and specialists. Given that more unique taxonomy was found in specialist SGBs, we also compared average estimated genome size and GC content of generalist and specialist in each phylum. Our result showed that, among various phyla, only the generalist SGBs from the Proteobacteria and Actinobacteriota exhibited a significant larger average estimated genome size than specialist SGBs (Wilcox rank sum test; both *p* value < 0.01), and only the generalist SGBs from Proteobacteria and Bdellovibrionota showed significant high GC content than specialist SGBs (Wilcox rank sum test; both *p* value < 0.01) (Additional file 2: Fig. S2). When removed the unique phyla that only found in specialist SGBs, we found generalist SGBs still had significant high larger average estimated genome size than specialist SGBs (Wilcox rank sum test; both *p* value < 0.01) (Additional file 2: Fig. S3).

### Various metabolic potentials of generalists and specialists in the cryoconite microbiome

**Fig. 2:**
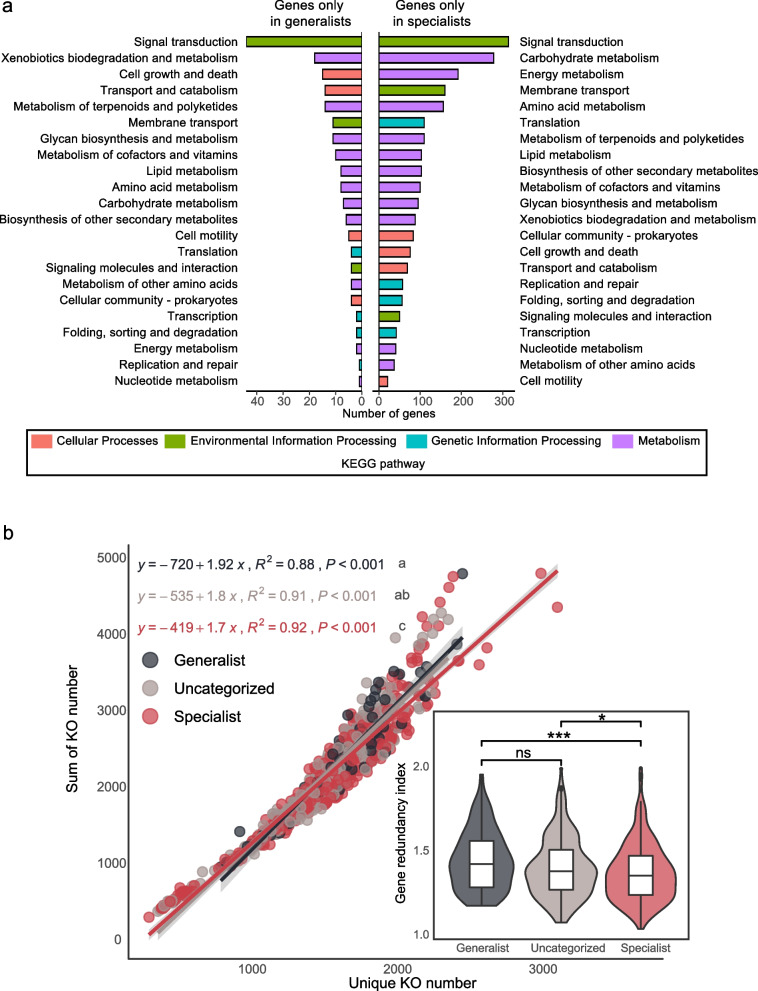
Unique function and gene redundancy in niche breath groups. **a** Quantification of genes annotated against KEGG database that were only found in generalists or specialists. **b** Linear regression fit between unique KO number and the sum of KO number, and the comparison of gene redundancy index among niche breadth groups in the sketch

Generalists and specialists have various potential functions. There were 115 and 1532 unique KEGG-annotated genes among the generalists and specialists, respectively (Fig. 2a). These genes could be classified into multiple functions or pathways based on the KEGG pathway categorization level. The top five functions (i.e., KEGG pathway level 2) of generalists belong to three pathways (i.e., KEGG pathway level 1), that is, environmental information processing, metabolism, and cellular process. A total of 15.6% of unique genes in generalist group were classified into metabolic pathways, including 8.7% involving xenobiotic biodegradation and metabolic functions and 6.8% involving terpenoid and polyketide metabolism functions. For the cellular process pathway, 7.3% of genes were associated with cell growth and death functions, and 6.8% were associated with transport and catabolism functions. The top five functions of specialists belong to two pathways, that is, metabolism and environmental information processing. A total of 25.9% of genes were classified into the metabolism pathway, including 11.5% involving carbohydrate metabolism, 7.9% involving energy metabolism and 6.5% involving amino acid metabolism. Another function in the environmental information processing pathway was membrane transport, which was associated with 6.5% of specialist unique genes. Signal transduction of environmental information processing was the most abundant function among both generalists and specialists, accounting for 21.5% of generalist and 13.8% of specialist unique genes, respectively.

Linear regression between unique KO number and sum of KO number showed that slope value in generalists was significantly larger than that in specialists (diffslope: *p* value < 0.001), which indicated that generalists had more duplicated genes than specialists **(**Fig. 2b**)**. To avoid genomic completeness bias, we also assessed the linear regression in different genome completeness categories. Generalists consistently exhibited higher slope values among different completeness categories (Additional file 2: Fig. S4). Furthermore, the ratio of unique KO numbers and the sum of KO numbers was used to quantify the degree of gene duplication of each SGB, which we named as the gene redundancy index. Our results showed that the gene redundancy index was significantly higher in generalists than specialists (Wilcox rank sum test; *p* value < 0.0001) (Fig. 2b).

### Biogeography of generalists and specialists across the Northern Hemisphere glacier cryoconite

**Fig. 3:**
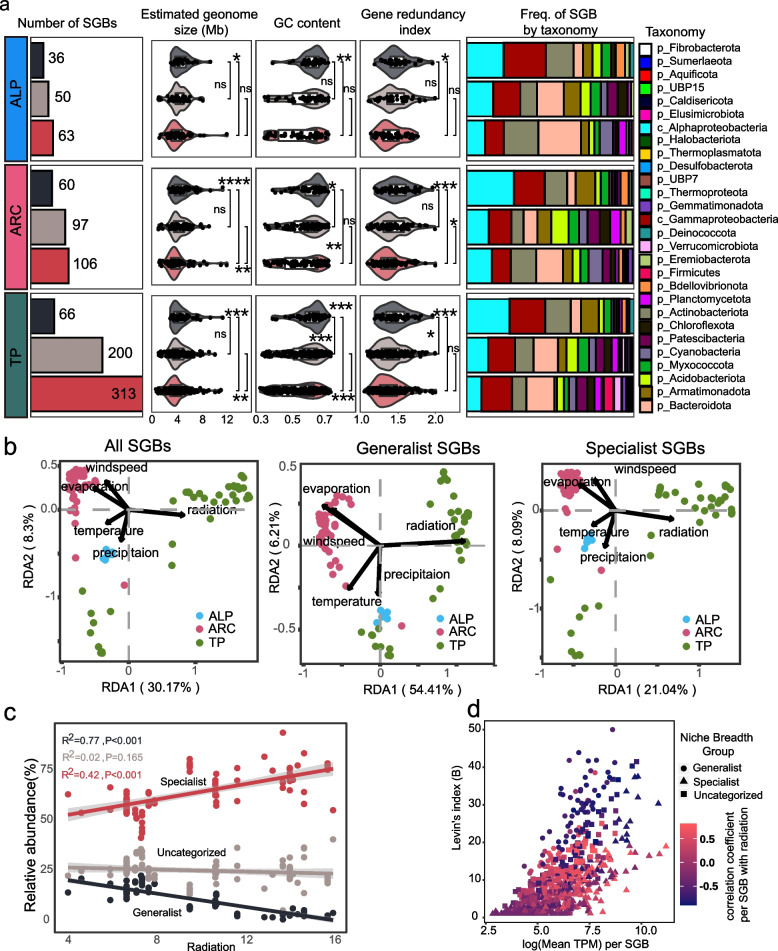
Biogeography of niche breadth groups and their relationship with meteorological factors. **a** Number of generalists, specialists, and uncategorized in Tibetan plateau (TP), Arcitc (ARC), and Alpine (ALP). The adjacent box plots show the difference in estimated genome size, GC content, and gene redundancy index within each subcategory. Adjacent stacked bar plots indicate their taxonomic composition at the phylum level (Proteobacteria phylum was at class level). **b** Relationship between community compositions and meteorological factors in all SGBs, generalist, and specialist based on redundancy analysis (RDA). All meteorological factors were tested in RDA analysis using the *envfit* function with 999 permutations. Significance level: **** (*p* ≤ 0.0001); *** (*p* ≤ 0.001); ** (*p* ≤ 0.01); * (*p* < 0.05). **c** Linear fit between the abundance of SGBs and radiation (MJ/${m}^{2}$) across niche breadth groups. **d** Correlation of each SGB between radiation and distribution of SGBs based on their logged mean read recruitments across cryoconite samples (X axis) and Levin’s Index (i.e., niche breadth, Y axis). The color gradient depicts Spearman’s correlation coefficient with radiation of each SGB and point shape indicates niche breadth groups

The number of generalists was smaller than that of specialists in all three biogeographical categories (i.e., ALP, ARC, and TP) across the Northern Hemisphere, but the differences were not the same (Fig. 3a). Although the number of SGBs in TP was twice as high as that in ARC and four times as high as that in ALP, the number of generalists was only one-fifth of the number of specialists in TP and one-half of that in ALP and ARC. For genome features, generalists had significantly larger genomes, GC contents, and gene redundancy index values than specialists in all three biogeographical categories (*p* value both < 0.05).

Generalists and specialists have similar dominant phyla in three biogeographical categories, that is, the phyla Proteobacteria, Bacteroidota, Actinobacteriota, and Armatimonadota. However, several groups showed different frequencies among biogeographical categories. For generalists, the frequency of the group Gammaproteobacteria in ALP was higher than that in other regions, and the groups Cyanobacteria and Patescibacteria were detected only in ARC and TP. For specialists, the frequency of the phylum Bacteroidetes was the highest in ALP, and those of the groups Firmicutes and Gammaproteobacteria were the highest in TP (Fig. 3a).

Clustering analysis showed optimum number of clusters was 10 according to k-mediod algorithm (Additional file 2: Fig. S5). Of these 10 clusters, samples of ALP were close to samples of Yala and Parlung No. 4 glacier in TP. Moreover, samples of ALP, ARC, and TP could approximately group into three more larger clusters in line with their geographic location. RDA results revealed that the whole, generalist, and specialist communities in ALP and ARC clustered together according to their geographical locations and were associated with meteorological factors (Fig. 3b). The whole, generalist, and specialist communities were all positively correlated with precipitation in ALP and wind speed in ARC (whole: *F* = 15.456, *p* value = 0.001; generalist: *F* = 35.198, *p* value = 0.001; specialist: *F* = 10.87, *p* value = 0.001). Most of the samples in the TP were positively correlated with radiation. In particular, nine samples from the Yala and Parlung No. 4 glacier was positively correlated with precipitation and closer to the ALP samples (Fig. 3b). In the whole community, the RDA results revealed that five meteorological factors, radiation, precipitation, temperature, windspeed, and evaporation, explained 45.4% of the variation in whole-community structure, whereas these factors explained 66.3% and 36.2% of the variation in the generalist and specialist communities, respectively. Among the five meteorological factors, the RDA and MRM analysis showed that radiation had a greater influence on the community structures than the other meteorological factors (Table 1). The contribution of radiation was the highest, accounting for 41.85% of the total differential contributions in the whole community, 44.60% in generalists and 39.77% in specialists. The standard partial regression coefficient of radiation was also the highest among meteorological factors, 0.80 in generalists, 0.78 in the whole community, and 0.75 in specialists (Table 1). Compared with geographic distance, radiation difference of each samples showed higher explanation (linear fitting: radiation difference, *R*^2^ = 0.59, *p* value < 0.001; geographic distance, *R*^2^ = 0.49, *p* value < 0.001) (Additional file 2: Fig. S6). MRM analysis also showed radiation had a greater influence on the whole community structures than geographic distance (standard partial regression coefficient: radiation difference = 0.82; geographic distance =  − 0.06) (Additional file 1: Table S3).

**Table 1:** Relative importance of each meteorological factor for community variation based on multiple regression analysis on matrices analysis (MRM) and redundancy analysis (RDA)

	All SGBs			Generalists			Specialists		
	MRM		RDA	MRM		RDA	MRM		RDA
	Standard partial regression coefficient	*p* value	Independent contribution (%)	Standard partial regression coefficient	*p* value	Independent contribution (%)	Standard partial regression coefficient	*p* value	Independent contribution (%)
Radiation	0.78	0.0001	41.85	0.80	0.0001	44.60	0.75	0.0001	39.77
Evaporation	0.05	0.0024	23.11	− 0.02	0.2337	25.67	0.08	0.0002	21.82
Windspeed	0.06	0.0007	14.03	0.03	0.0997	12.64	0.09	0.0001	15.01
Precipitation	− 0.04	0.0247	8.28	− 0.02	0.2010	5.08	− 0.03	0.1385	10.61
Temperature	0.10	0.0002	12.65	0.14	0.0001	11.98	0.08	0.0013	12.77

Generalists and specialists showed contrasting correlations with radiation, as indicated by linear correlation analysis (Fig. 3c). The accumulative abundance of generalists and specialists significantly declined (*R*^2^ = 0.77, *p* < 0.001) and increased (*R*^2^ = 0.42, *p* < 0.001) with increasing radiation, respectively. In addition, the diversity of generalists and specialists indicated by the cumulative number of SGBs was negatively and positively correlated with radiation (*R*^2^ = 0.77, *p* < 0.001; *R*^2^ = 0.43, *p* < 0.001), respectively (Additional file 2: Fig. S7). However, not all generalists were negatively correlated with radiation, nor were all specialists positively correlated with radiation. Spearman’s test showed that 536 SGBs were significantly correlated with radiation, including 70 generalists and 282 specialists (both *p* value < 0.05). A total of 72% of generalists and 50% of specialists were negatively correlated with radiation (Fig. 3d). Moreover, the absolute value of Spearman’s correlation coefficient of generalists was significantly higher than that of specialists (Wilcox rank sum test; *p* value < 0.0001) (Additional file 2: Fig. S8), indicating that generalists were more sensitive to radiation than specialists.

### The linkage between ubiquitous functions of community and niche breadth groups

**Fig. 4:**
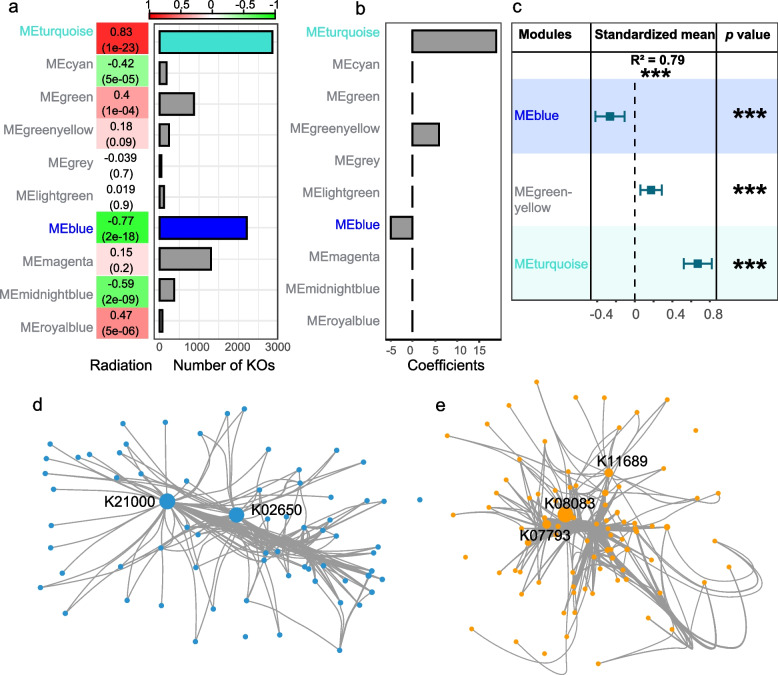
Co-occurrence networks of metagenomic KEGG Orthologs.** a** Correlation heatmap between the network modules and radiation. The colors correspond to the correlation values, red is positively correlated, and blue is negatively correlated. The values in each of the squares correspond to the assigned Pearson correlation coefficient value on top and *p*-value in brackets below. Adjacent barplots indicate KEGG Orthologs (KOs) numbers of each module. **b** Relative importance of network modules for predicting radiation variation based on L1-regularized regression that least absolute shrinkage and selection operator (LASSO). **c** Results of multiple regressions after selection process of LASSO on all modules. Each variable was standardized before comparing effect sizes (squares) to determine differences in the strength of predictor variables. The error bars represent 95% confidence intervals (CI) and indicate a significant (*p* < 0.05) effect when not overlapping with 0. **d** Co-occurrence network of selected genes involving biofilm formation in module MEblue. **e** Co-occurrence network of selected genes involving biofilm formation in module MEturquoise. Node size represents betweenness centrality, and high betweenness centrality nodes are labeled their KOs

Owing to the gap in genome functions and community functions, we further analyzed the ubiquitous function of the community in response to radiation stress by a gene-centric approach. A total of 8272 KOs were found in at least 70% of samples, which could be prevalent functions of the cryoconite microbiome, and these KOs clustered into 10 function modules in network analysis (Fig. 4a). Among these modules, the gene number of MEturquoise (2858 KOs) was highest, followed by that of MEblue (2210 KOs). Moreover, over half of the 10 modules were significantly correlated with radiation (Pearson’s correlation test; both *p* value < 0.05), except for MEmagenta, MElightgreen, MEgreenyellow, and MEgrey. Three modules were negatively correlated with radiation, and the MEblue module exhibited the highest Pearson’s correlation coefficient (− 0.77; *p* value = 7e − 18). In contrast, three modules were positively correlated with radiation, and the MEturquoise module exhibited the highest Pearson’s correlation coefficient (0.83; *p* value = 1e − 23).

LASSO regression analysis showed that three modules correlated with radiation under the best penalty (best lambda). MEblue had the highest negative correlation coefficient, and MEturquoise had the highest positive correlation coefficient (Fig. 4b). These three modules explained almost 79% of the variation in radiation (*R*_adj_ = 0.785, *p* value < 0.001) based on the standardized regression coefficient approach. Among these three modules, the MEblue and MEturquoise modules explained the greatest amounts of variation in radiation (Fig. 4c).

**Fig. 5:**
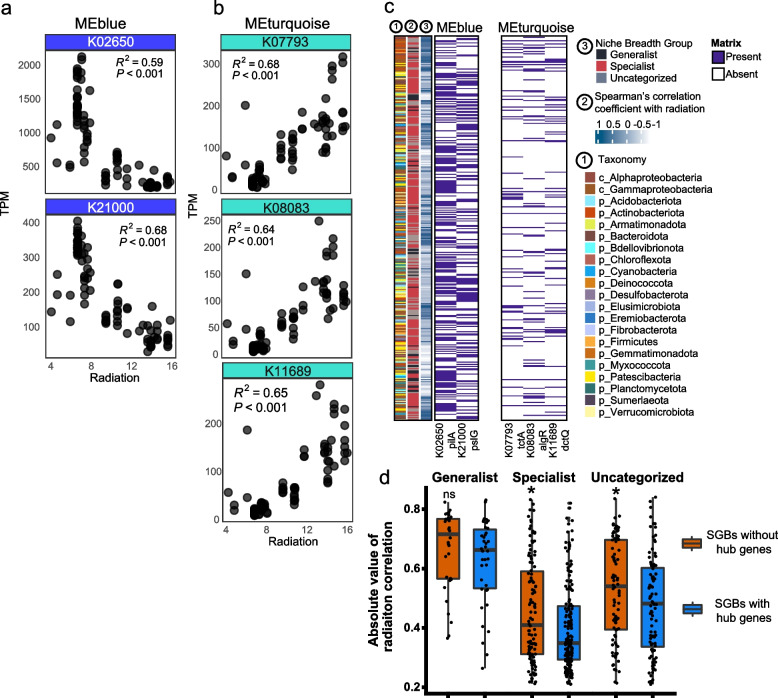
The hub genes related to radiation in MEblue and MEturquoise and their distribution across niche breadth groups. **a** Linear regression fit between each hub gene and radiation (MJ/${m}^{2}$) in MEblue. **b** Linear regression fit between each hub gene and radiation (MJ/${m}^{2}$) in MEturquoise. **c** Distribution of hub genes among niche breadth groups. **d** Comparing the correlation of species to radiation in the presence or absence of hub genes (Wilcox rank sum test). Significance level: **** (*p* ≤ 0.0001); *** (*p* ≤ 0.001); ** (*p* ≤ 0.01); * (*p* < 0.05)

We further identified representative pathways of each function module by the clusterProfiler enrichment approach (Additional file 1: Table S4). Given that MEblue and MEturquoise modules had strongest correlation than other modules, we selected these two modules to find hub genes accented with radiation variation. In module MEblue, most of the genes (91 KOs) were enriched in the two-component system pathway, followed by the function unknown pathway (70 KOs), bacterial motility protein pathway (52 KOs) and quorum sensing (52 KOs) (Additional file 1: Table S4; Additional file 2: Fig. S9a). In module MEturquoise, most of the genes (222 KOs) were in two-component system pathways, followed by ABC transporters (116 KOs) and unknown function pathways (100 KOs) (Additional file 1: Table S4; Additional file 2: Fig. S9b). The biofilm formation pathway was enriched in module MEturquoise and bacterial motility pathway was enriched in module MEblue. Radiation-related hub genes were also associated with bacterial motility proteins, biofilm formation, flagellar assembly, O-antigen nucleotide sugar biosynthesis, quorum sensing, secretion system, and two-component system (Additional file 1: Table S4). Genes of these pathways were connected by five hub genes based on network analysis (Fig. 4d–e): two hub genes (K21000 *pslG*; K02650 *pilA*) in MEblue and three hub genes (K11689 *dctQ*; K08083 *algR*; K07793 *tctA*) in MEturquoise. Further analysis showed that all five hub genes were significantly correlated with radiation (linear model fitting, *R* = 0.59 ~ 0.68, *p* value < 0.001) (Fig. 5a–b). The *pslG* (K21000) gene had the strongest negative correlation with radiation among the hub genes and explained 68% of the radiation variation; in contrast, the *tctA* (K07793) gene had the strongest positive correlation with radiation among the hub genes and explained 68% of the radiation variation.

Given that the accumulative abundance of generalists and module’s eigengene values of MEblue had similar negative correlations with radiation, in contrast, specialists and MEturquoise had similar positive correlations with radiation (Figs. 3c and 4a), we queried the sequences of five hub genes from 645 SGBs to link cryoconite taxa and the ubiquitous function that related to radiation of the community (Fig. 5c). In total, over half of the SGBs (54%) contained at least one type of hub gene sequence. These SGBs distributed into majority of bacterial phyla, except for phyla Aquificota, Caldisericota, UBP15 and UBP7. Most of these SGBs were most frequently assigned to the phylum Proteobacteria (30%), followed by the Patescibacteria (9%) and Armatimonadota (8%). However, these hub genes were not detected in any archaeal SGBs. Hub genes from different modules could be found simultaneously in one SGB. For instance, four hub genes were simultaneously detected in 12 SGBs belonging to the Gammaproteobacteria class. The genera Rhodoferax and Rhizobacter (members of the family Burkholderiaceae), which are associated with biofilms in other ecosystems [68, 69], accounted for approximately 42% of these SGBs. When compared to SGBs without hub genes, our results showed that specialist SGBs with hub genes had significantly lower absolute values of Spearman’s correlation coefficient with radiation (Wilcox rank sum test; *p* value < 0.05) (Fig. 5d).

## Discussion

### Radiation influences specialists and generalists

Low temperature and oligotrophy are common features of glacial ecosystems, and the presence of radiation gradients may greatly influence the cryoconite microbiome. In this study, we investigated the relationship between five meteorological factors (i.e., solar radiation, temperature, precipitation, wind, and evaporation) and the cryoconite microbiome and highlighted the influences of radiation on generalists and specialists in cryoconite. Our results showed that the accumulative abundance and species number of generalists in the community decreased with increasing radiation, while the opposite trend was observed in specialists (Fig. 3c; Additional file 2: Fig. S7). This result suggested that radiation could influence community turnover and composition. Generalists are often adapted to various habitats [70], while specialists are highly adapted to a single environment [19]. In a previous study in coastal Antarctic lakes, abundance of habitat specialists was also found to increase along salinity gradients [71]. Therefore, it is likely that environmental stress reduced proportion of generalists in community by influencing their dispersal strategy [72]. In general, the environmental stress causes species to cluster, whereas interspecific competition makes them disperse [73], probably due to cooperative communities have advantage in stress resistance. Indeed, cooperation among communities has significant biological advantages for individual members (e.g., maintaining complex community structures, increasing community productivity, and improving stress resistance) [74].

The differences between generalists and specialists in genome features and functions provide complementary evidence to explain their adaptation under radiation stress. Generalists had significantly larger genomes, GC contents, and gene redundancy index values than specialists in our study (Figs. 1 and 2). In each phylum, generalists exhibited larger genomes, higher GC contents, and greater gene redundancy index values compared to specialists. However, a statistically significant difference of these features between generalists and specialists was only observed in the phyla Proteobacteria, Bdellovibrionota, and Actinobacteriota (Additional file 2: Fig. S2). This result suggested that taxonomic differences between generalists and specialists should be considered when comparing genomic features and gene redundancy index. Nevertheless, after excluding specialist unique phyla, it was observed that generalists also had significantly larger genomes, GC contents, and gene redundancy index values than specialists (Additional file 2: Fig. S3). Genome sizes are usually positively correlated with cell sizes, which determine the surface-to-volume ratio, with smaller cells benefitting nutrient acquisition due to the increased surface-to-volume ratio [75]. On the other hand, species with large genome sizes could have more genes and a larger proportion of redundant genes [76]. Gene redundancy contributes to genomic robustness [77], which allows genes loss or mutation, and helps maintain stability after environmental perturbation [78]. Generalists with higher gene redundancy are likely to have higher metabolic flexibility in order to fit broader habitats. Specialists with lower gene redundancy are likely to adapt to extreme conditions by improving energy utilization efficiency. The difference in gene redundancy between generalists and specialists suggested that the species could adapt specific habitat by avoiding gene redundancy through redundant gene loss and evolution. Redundant gene evolution could contribute to product new function in environment adaption [79]. Gene loss is a general evolutionary mechanism that maintain reasonable genome size and improves energy utilization efficiency [80]. This is consistent with the “black queen” hypothesis [81] that free-living organisms in the community adapt to the environment through gene loss and the use of common resources in the community [82]. In addition, generalists had significantly higher GC contents than specialists (Fig. 1b). Given that Bourquin et al. (2022) revealed that cryospheric genera had higher GC contents than other genera [83], it seems that cryoconite generalists are more likely to adapt to cold environments, whereas specialists could be restricted by other environmental factors, such as thermoacidophilic archaea (*Metallosphaera sedula*), which are found only in the specialist group [84].

In addition to different cryoconite species, community structure was also influenced by radiation (Fig. 3b). Radiation as a primary energy source not only supports phototrophic microorganisms but also, as an environmental stressor, causes clear selection pressure [85, 86]. Previous studies have also shown that radiation impacts community structure in natural ecosystems, such as the ocean [87] and meadows [88]. Indeed, RDA revealed that TP samples were more related to radiation (Fig. 3b) because the TP region is exposed to more radiation than the Arctic owing to its higher altitude and lower latitude. Within the TP region, major samples were more related to radiation, but samples from the Yala and Parlung No. 4 glaciers were more related to precipitation. Murakami et al. also reported that Yala glacier samples were separated from central Asian glaciers when comparing community structure [89]. The Yala glacier is located on the southern slope of the Himalayan Mountains, and the Parlung No. 4 glacier is located southeast of the Tibetan Plateau. Moreover, the Yala and Parlung No. 4 glaciers are influenced by monsoons, which may bring more precipitation than received by other glaciers in the TP region [90]. This result suggests that community structure is influenced by regional climate. Nevertheless, radiation showed a more significant influence on community similarity than other meteorological factors (Table 1). One plausible reason is that radiation is more stable than other meteorological factors and persistently impacts the dynamics of Northern Hemisphere glacier cryoconite. Furthermore, meteorological factors had greater influences on generalist community variation than specialist and whole community variation (Fig. 3b), suggesting that the presence of specialists could enhance community stability by increasing functional redundancy and microbial interactions. This phenomenon was comparable to that described for the endosymbiotic coral system, where generalists showed greater susceptibility to environmental stress than specialists [91]. Hence, distinguishing the role of species in communities is meaningful for predicting the dynamics of ecosystems under environmental change.

### Radiation influences microbial biofilm formation in cryoconite

Unlike gene redundancy in single taxa (i.e., multicopy gene in a genome), distinct taxa also perform the same function, known as functional redundancy [92]. These same functions represent the prevalent functional groups of the community adapted to a given environment. Network analysis based on the prevalent function of community identified two distinct network modules (i.e., identical functional groups) that had strong relationships (positive and negative correlations) with radiation (Fig. 4). Both modules were associated with biofilm formation, which could be a ubiquitous function in response to radiation stress. Biofilms have been found in diverse environments and have proven to be ecologically advantageous for survival [93]. Moreover, biofilms in glacier ecosystems contribute to microbe colonies and act as physical barriers against extreme environmental conditions (e.g., high UV radiation and low temperature) [10, 94, 95]. Previous studies on cryoconite microorganisms identified EPS of cyanobacteria as one of the driving forces for cryoconite biofilm and aggregation formation, which contributes to adaptation to low temperature, strong radiation, and oligotrophic environments [7, 8]. Under light conditions, increased excretion of EPS was observed in an incubated unicyanobacterial biofilm [14]. However, at the community level, the relationship between cryoconite microbes and biofilms has not been revealed under various radiation stress conditions. Our results showed that specialists with any hub genes had weaker correlations with radiation than those without hub genes, suggesting that biofilm regulation could alleviate the effects of radiation (Fig. 5d). Hence, understanding the microbial biofilm lifestyle at the community level is meaningful for investigating the survival strategy of communities under radiation stress.

**Fig. 6:**
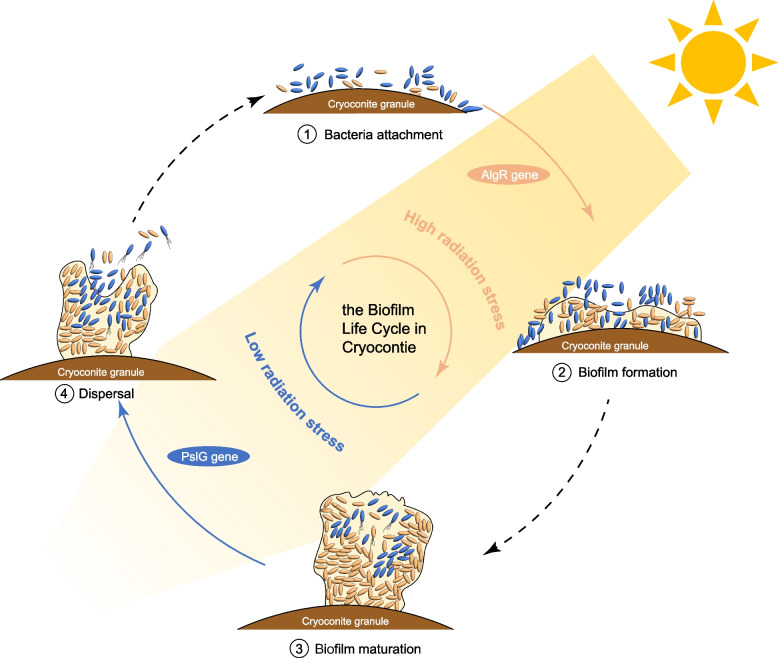
Hypothesized “stay or escape” survival strategy of cryoconite microbiomes in response to radiation stress. Biofilm regulations under high radiation are in orange, while biofilm regulations under low radiation stress are in blue

The network and clustering analyses showed that the cryoconite microbial community had two opposing biofilm patterns (formation and dispersion) that corresponded to various radiation intensities, and each of the patterns was controlled by different hub genes. Specifically, hub genes (i.e., *dctQ*, *algR* and *tctA*) related to biofilm formation were positively correlated with radiation (Fig. 5b), while genes (i.e., *pslG* and *pilA*) related to biofilm dispersion were negatively correlated with radiation (Fig. 5a). It is suggested that cryoconite microbes may follow a “stay or escape” survival strategy in response to radiation stress variation (Fig. 6). Under high radiation stress, microbes tend to stay in the biofilm matrix to avoid radiation damage. Three hub genes of MEturquoise were all related to biofilm formation: *dctQ*, *algR* [96], and *tctA*. The *dctQ, dctM*, and *dctP* genes encode the C_4_‐dicarboxylate tripartite ATP‐independent periplasmic (TRAP) transporter large permease protein and transport C_4_‐dicarboxylate into the cytoplasm and be used as a carbon and energy source in bacteria [97, 98]. The *dctP* gene was reported to regulate the colonization, adhesion, and pathogenicity of *Vibrio alginolyticus* strain HY9901 [99]. The *algR* gene regulates the production of the polysaccharide alginate and type IV pilus-mediated twitching motility [100], which is important for EPS and biofilm formation. The *tctA* gene encodes tricarboxylate transport protein, associated with the two regulatory components *tctD* and *tctE* that regulate the uptake of tricarboxylic acids and influence biofilm development [101, 102]. Under low radiation stress, microbes tend to escape from biofilms to acquire more nutrients and harbor dispersal-related genes, such as the *pslG* [103]. The *pslG* gene had the strongest negative correlation with radiation, and it can disrupt the Psl extracellular polysaccharide matrix to prevent biofilm formation [103]. In general, *pilA* gene is associated with pili formation, which contributes to cell motility [104]. A pervious study in *Pseudomonas aeruginosa* biofilms had reported that higher lower expression level of *pilA* gene in planktonic and dispersed cells [105]. However, the relationship between radiation and cryoconite ecosystem could be more complex than this hypothesized model. In addition, in model strains (e.g., *Deinococcus swuensis* Strain DY59^T^ and *Sphingomonas* sp. strain UV9) studies [106, 107], DNA repair gene expression was the main way to cope with radiation stress [108]; however, in our study, no clear correlation between DNA repair genes and radiation was found, probably due to biofilm formation largely preventing radiation damage to DNA.

These hub genes were detected in the majority of SGBs (Fig. 5c), further supporting that biofilm-related functions widely exist in the cryoconite community. Given that hub genes have multiple functions and may be involved in other fundamental pathways, such as cell motility and nutrient transport, the number of species engaged in biofilm regulation might be overestimated. Indeed, a study of Antarctic cryoconite found that nearly 35% of the cryoconite sediment surfaces were covered by biofilm as revealed by confocal laser scanning microscopy [13]. Our results showed the great biofilm regulation potential of cryoconite species, but we need to confirm this result in future research. On the other hand, it is not possible from the present results to establish a linkage between biofilm lifestyles and niche breadth groups (Fig. 5c). Although hub genes and niche breadth groups were correlated with radiation, horizontal gene transfer and genome completeness bias inevitably interfere with the connection between hub genes and niche breadth groups. Nevertheless, SGBs without hub genes were more sensitive to radiation variation, supporting that biofilm-associated functional regulation is important for cryoconite species adaptation to radiation stress (Fig. 5d). Furthermore, there was a likely linkage between biofilm lifestyle and microbial interactions in the cryoconite (Fig. 6). Under high radiation stress, the proportion of generalists is lower, and microbial cooperation could increase. Microbes stay in the biofilm matrix to improve resource use efficiency (use public goods and mutualism). Under low radiation stress, the proportion of generalists is higher, and microbial competition could increase. Microbes tend to escape from the biofilm matrix to seek diverse nutrients. In general, microbial interactions may be stronger in mature biofilms and contribute to more frequent nonspecific DNA uptake, preserving the possibility of new functional acquisition via horizontal gene transfer [109–111]. In summary, biofilms, as an emergent form of microbial life, are an efficient system for investigating cooperation, resource capture and survival strategies of the community in cryoconite.

## Conclusion

With the development of sequencing and bioinformatics, the study of microbial functional variation in cryoconite is currently available. In this study, we recovered 2078 medium–high quality genomic bins and built a nonredundant gene set within 17,008,994 nonredundant open reading frames. These data collectively constitute a large fraction of the cryoconite microbial diversity detected by metagenomics. We investigated cryoconite dynamics by distinguishing the roles of specialists and generalists and detected their connection with solar radiation. Our results highlight the gene redundancy of cryoconite species and biofilm formation regulation as key in the response to radiation stress. Collectively, our findings provide new insight into the cryoconite microbiome survival strategy in response to radiation stress and serve as a baseline for future monitoring of the state of cryoconite ecosystems.

### Supplementary Information

**Additional file 1.** Dataset Table 1–4.

**Additional file 2. **Dataset Figures.

## Data Availability

The datasets analyzed during the current study are available in the NCBI repository in the accession number of projects PRJEB12327, PRJNA283341, PRJNA360211, PRJNA560154, PRJDB11497, and PRJNA813429. All 88 assembled contigs, 17,008,994 nonredundant ORFs, and 645 SGBs genome have been uploaded in figshare.com (10.6084/m9.figshare.22357891, 10.6084/m9.figshare.22359100, and 10.6084/m9.figshare.22359037). Workflow of metagenomic data analysis have all been included as Additional file 2. Corresponding taxonomic classifications, genome features, and niche breadth groups of 645 SGBs have all been included as Additional file 1.

## Abbreviations

ANI: Average nucleotide identity
CI: Confidence interval
CLR: Centered log ratios
CPR: Candidate phyla radiation
GTDB: Genome Taxonomy Database
ECMWF: European Centre for Medium–Range Weather Forecasts
KEGG: Kyoto Encyclopaedia of Genes and Genomes Orthology
KO: KEGG Ortholog
LASSO: Least Absolute Shrinkage and Selection Operator
MAG: Metagenome assembly genome
MRM: Multiple regression on matrices
NCBI: National Center of Biotechnology Information
NetCDF: Network Common Data Form
ORF: Open reading frame
RDA: Redundancy analysis
SRA: Sequence Read Archive
SGB: Species-level genome bin
TRAP: ATP‐independent periplasmic
VAMB: Variational autoencoders for metagenomic binning
VIF: Variance inflation factor
WGCNA: Weighted gene co-expression network analysis
