# Supplementary material for: Radiation impacts gene redundancy and biofilm regulation of cryoconite microbiomes in Northern Hemisphere glaciers
Source: Microbiome. 2023 Oct 18;11:228. doi: 10.1186/s40168-023-01621-y (PMC10583317; doi:10.1186/s40168-023-01621-y)
Supplement: Supplementary file 3 — Additional file 2. Dataset Figures. [file 40168_2023_1621_MOESM2_ESM.docx]

# Supplementary Figure

## Supplementary Figure S1


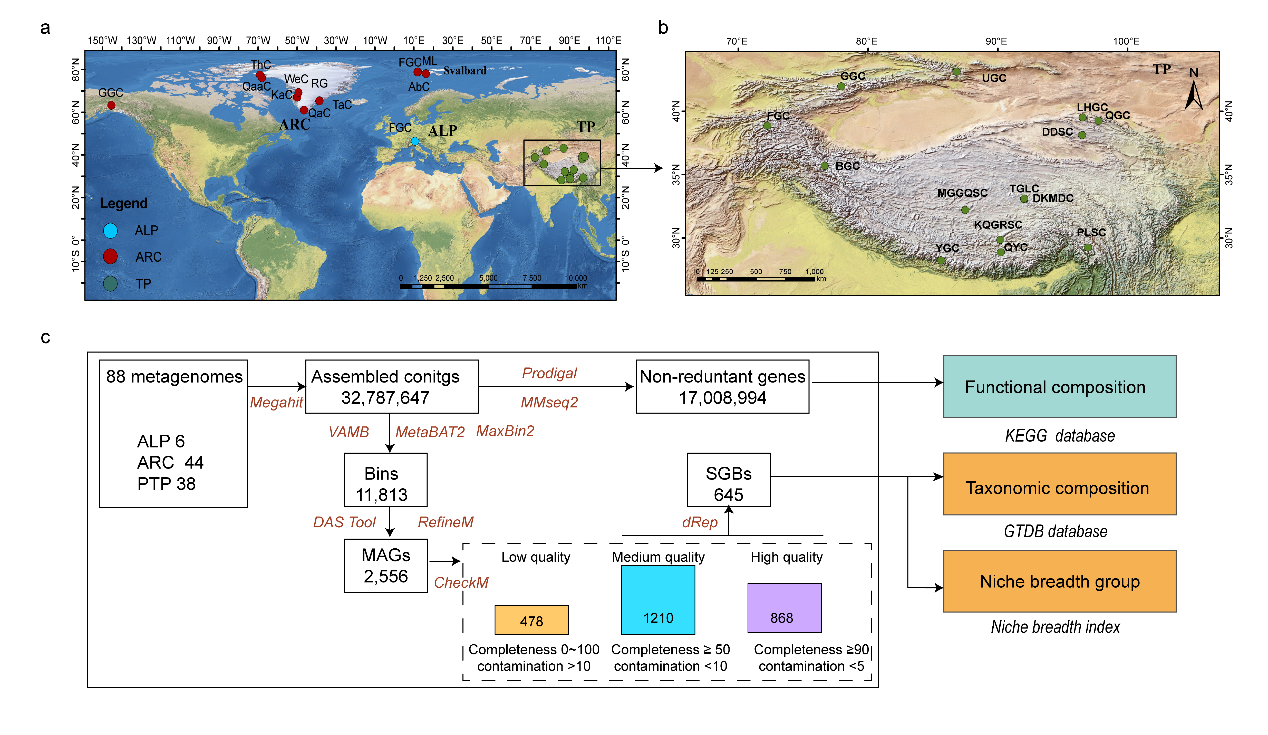


**Supplementary Figure S1. Sampling sites and metagenomic data workflow of northern hemisphere glacier cryoconite microbiome.** a, 88 metagenomes distribution of 26 sampled sites are shown based on the main glacial geography region, all samples are divided into three groups, ALP (samples from Alp), ARC (samples from the Arctic) and TP ( samples from Tibetan Plateau as well as surrounding regions). b, detail sampled sites in TP. c, Outline of northern hemisphere glacier cryoconite microbial metagenomic data, including the number of contigs, genes, and bins. All bins were refined by DAS Tools and refineM to obtain MAGs, and medium-high quality MAGs were clustered into SGBs based on ANI threshold 95. All softs were showed by brown italics.

## Supplementary Figure S2


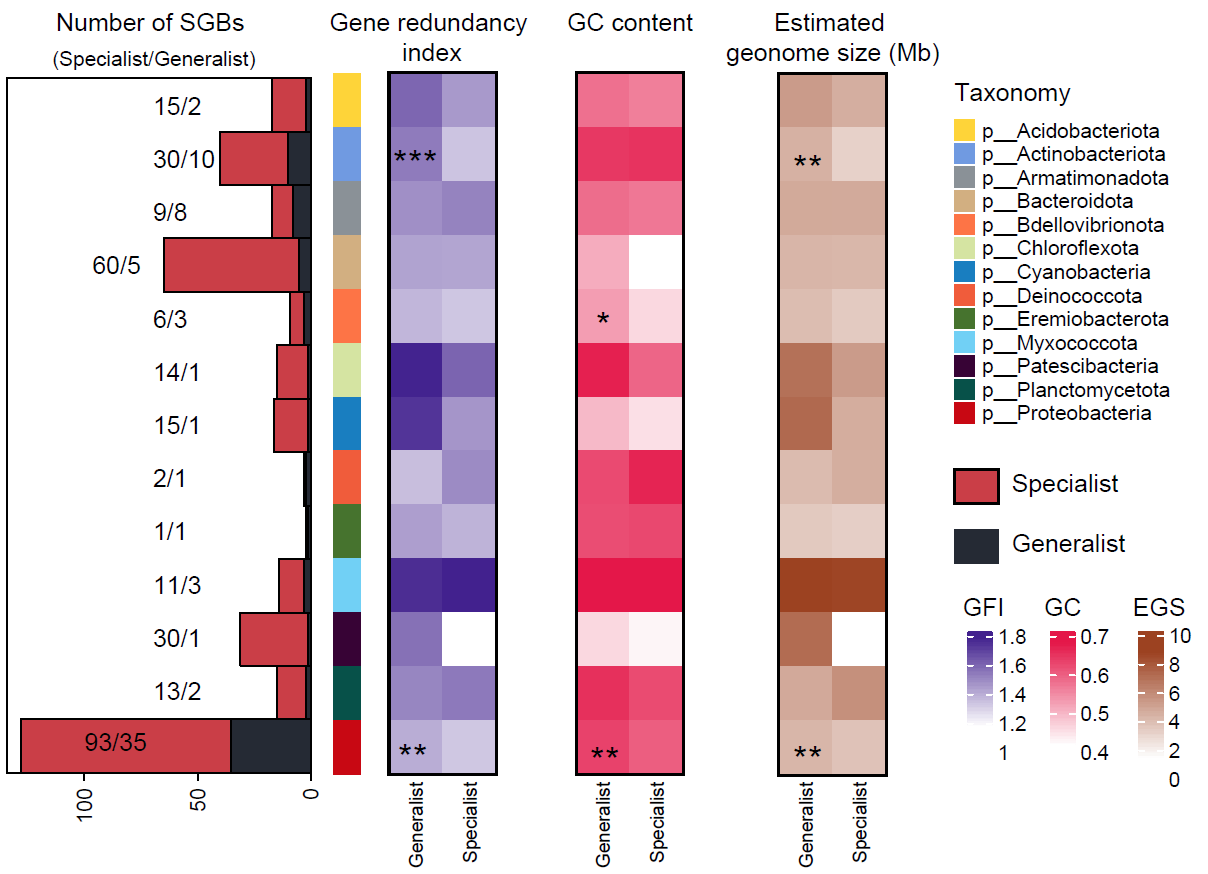


**Supplementary Figure S2. Genomic features and gene redundancy index difference between generalist and specialist SGBs in each phylum.** 13 phyla were simultaneously found in generalists and specialists. The left barpot shows number of generalist and specialist SGBs in 13 phyla. Adjacent heatmaps indicate the comparisons of gene redundancy index (GRI), GC content, and estimated genome size (EGS) between generalist and specialist SGBs (*Wilcox Rank Sum test*). Significance level: **** (p ≤ 0.0001); *** (p ≤ 0.001); ** (p ≤ 0.01); * (p < 0.05).

## Supplementary Figure S3


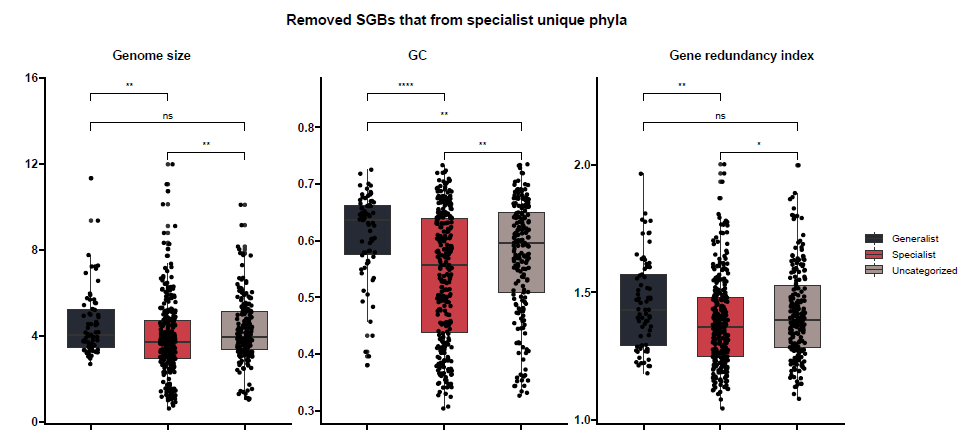


**Supplementary Figure S3. Genomic features and gene redundancy index difference between generalist and specialist SGBs when removed SGBs that from specialist unique phyla.** Statistic significances were calculated by *Wilcox Rank Sum test*. Significance level: **** (p ≤ 0.0001); *** (p ≤ 0.001); ** (p ≤ 0.01); * (p < 0.05).

## Supplementary Figure S4


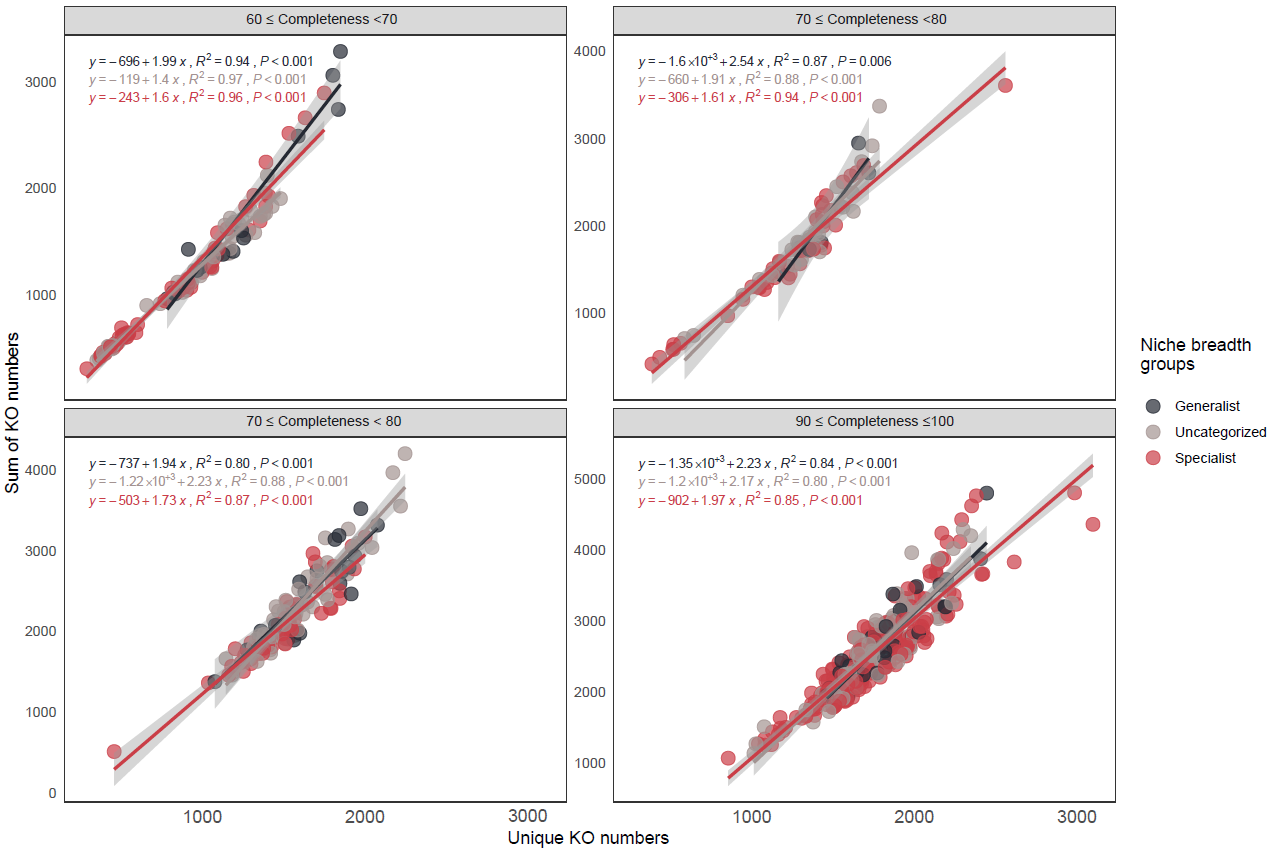


**Supplementary Figure S4.** **Linear correlations of unique KO numbers and sum of KO numbers under different completeness.** Owing to the lack of enough genomes, lower than 60 completeness SGBs were not shown.

## Supplementary Figure S5


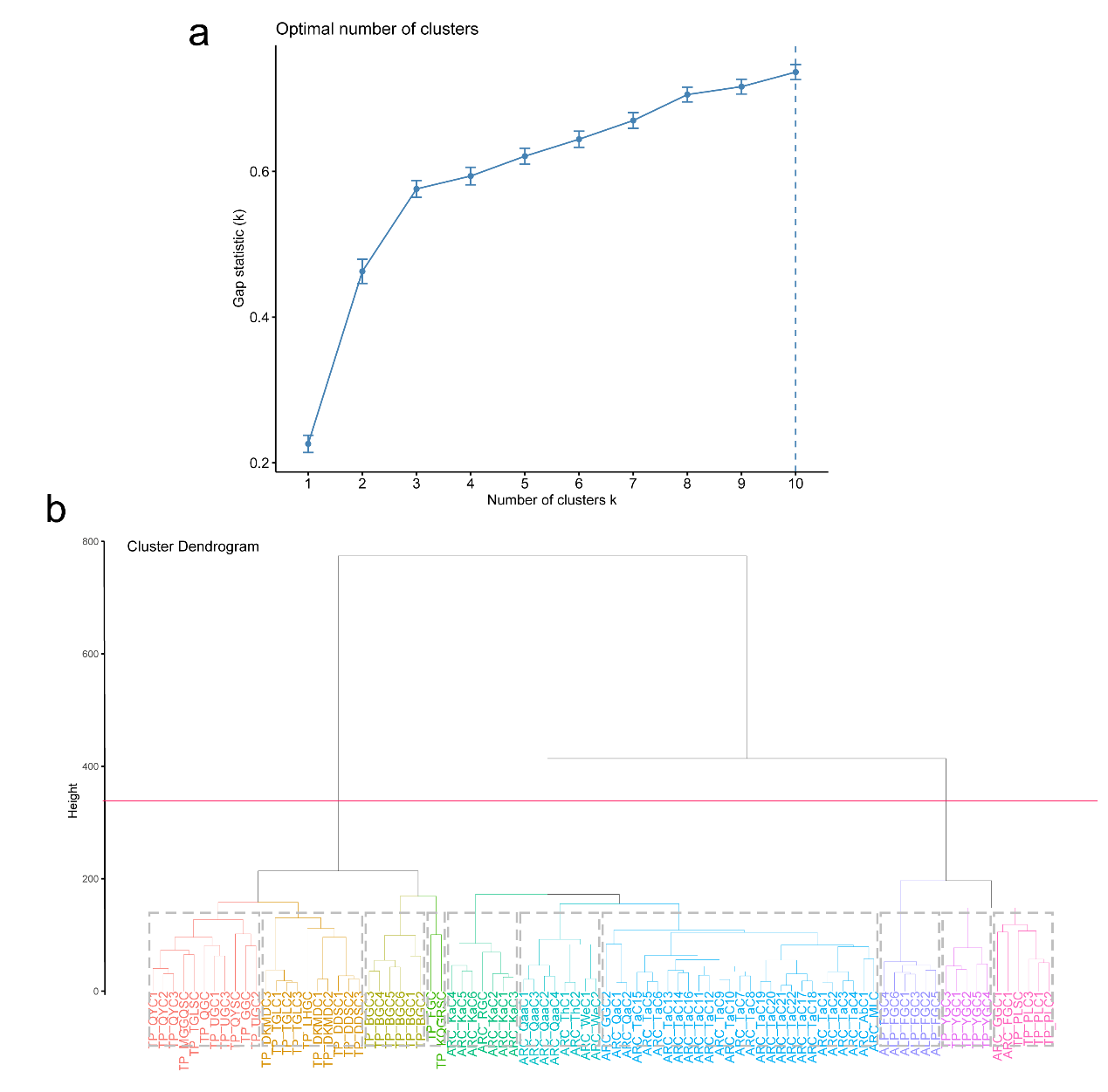


**Supplementary Figure S5. Clustering analysis of 88 cryoconite metagenomes.** a, The optimum number of clusters was determined by the gap statistic method using the function*fviz_gap_stat.* b, The dendrograms were plotted using the *fviz_dend* functions under optimum number of clusters. Red horizontal line emphasizes the three large cluster.

## Supplementary Figure S6


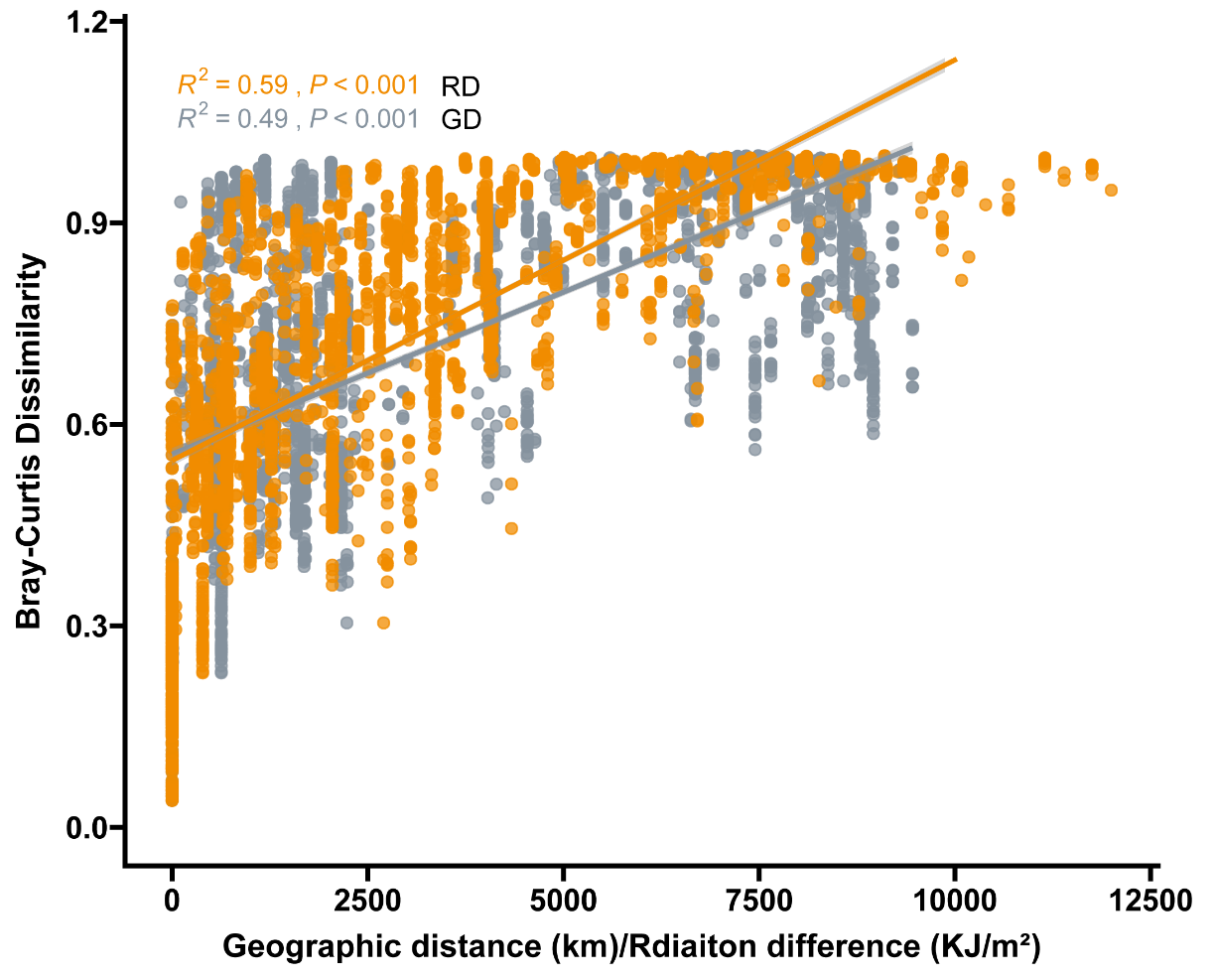


**Supplementary Figure S6. Linear relationships of community dissimilarity (Bray–Curtis index) with increasing geographical distance/radiation difference.** The regression lines were fitted to the data points using linear model. statistical information was labelled by *stat_poly_eq* function in the ggpmisc package in R. abbreviation: RD, radiation difference; GD, geographical distance.

## Supplementary Figure S7


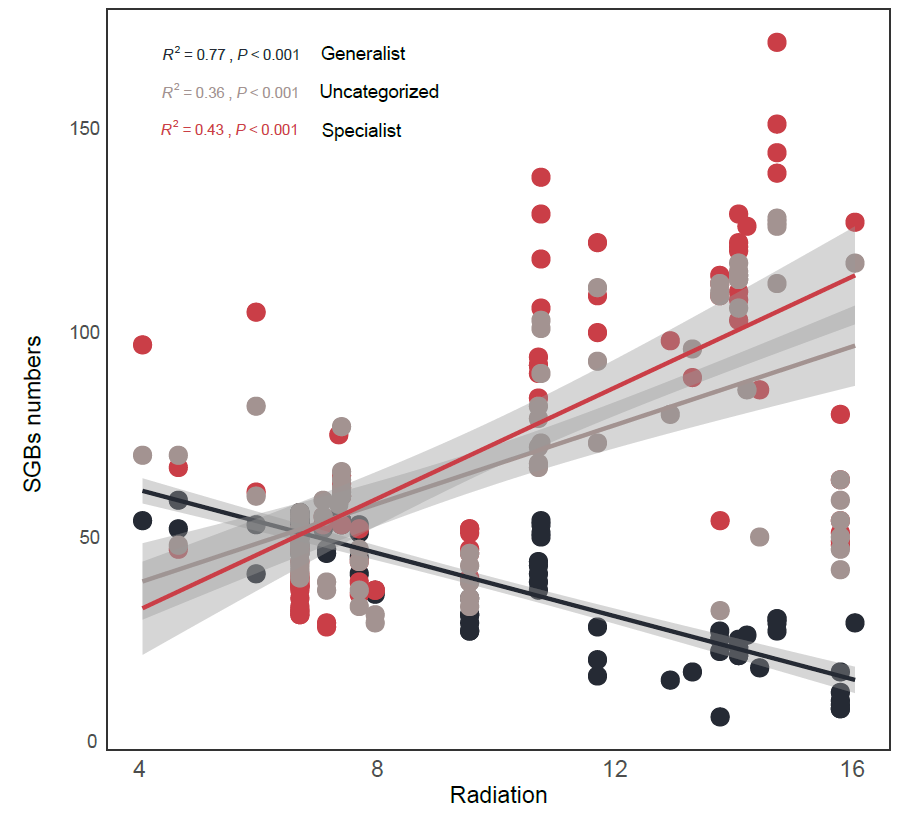


**Supplementary Figure S7. Linear fit between numbers of SGBs and radiation (MJ/m^2^) of niche breadth groups.**

## Supplementary Figure S8


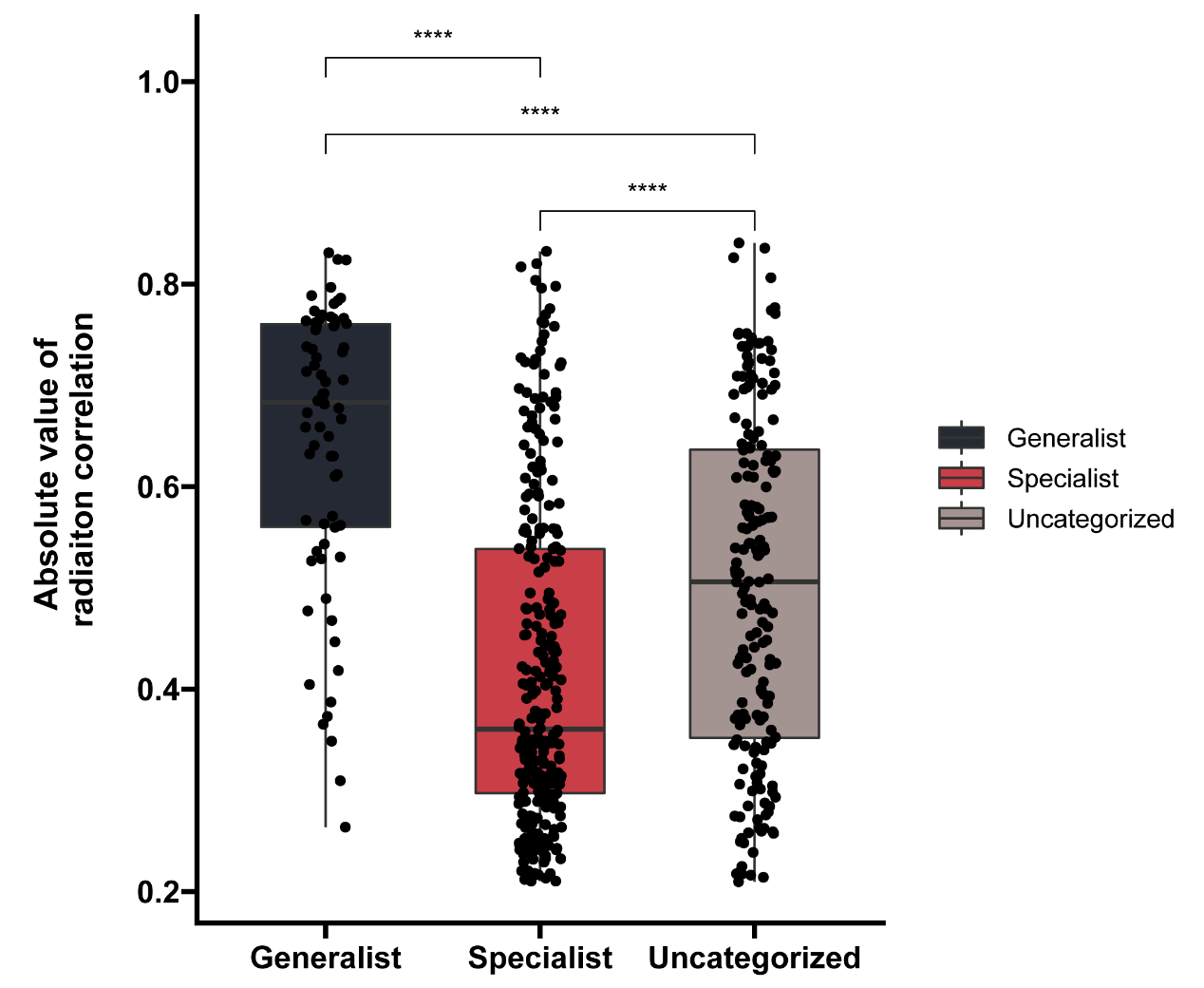


**Supplementary Figure S8. Comparison of radiation correlation among niche breath groups.** The radiation correlation was reflected by the Spearman’s correlation coefficient. Statistic significances were calculated by *Wilcox Rank Sum test*: **** (p ≤ 0.0001); *** (p ≤ 0.001); ** (p ≤ 0.01); * (p < 0.05).

## Supplementary Figure S9


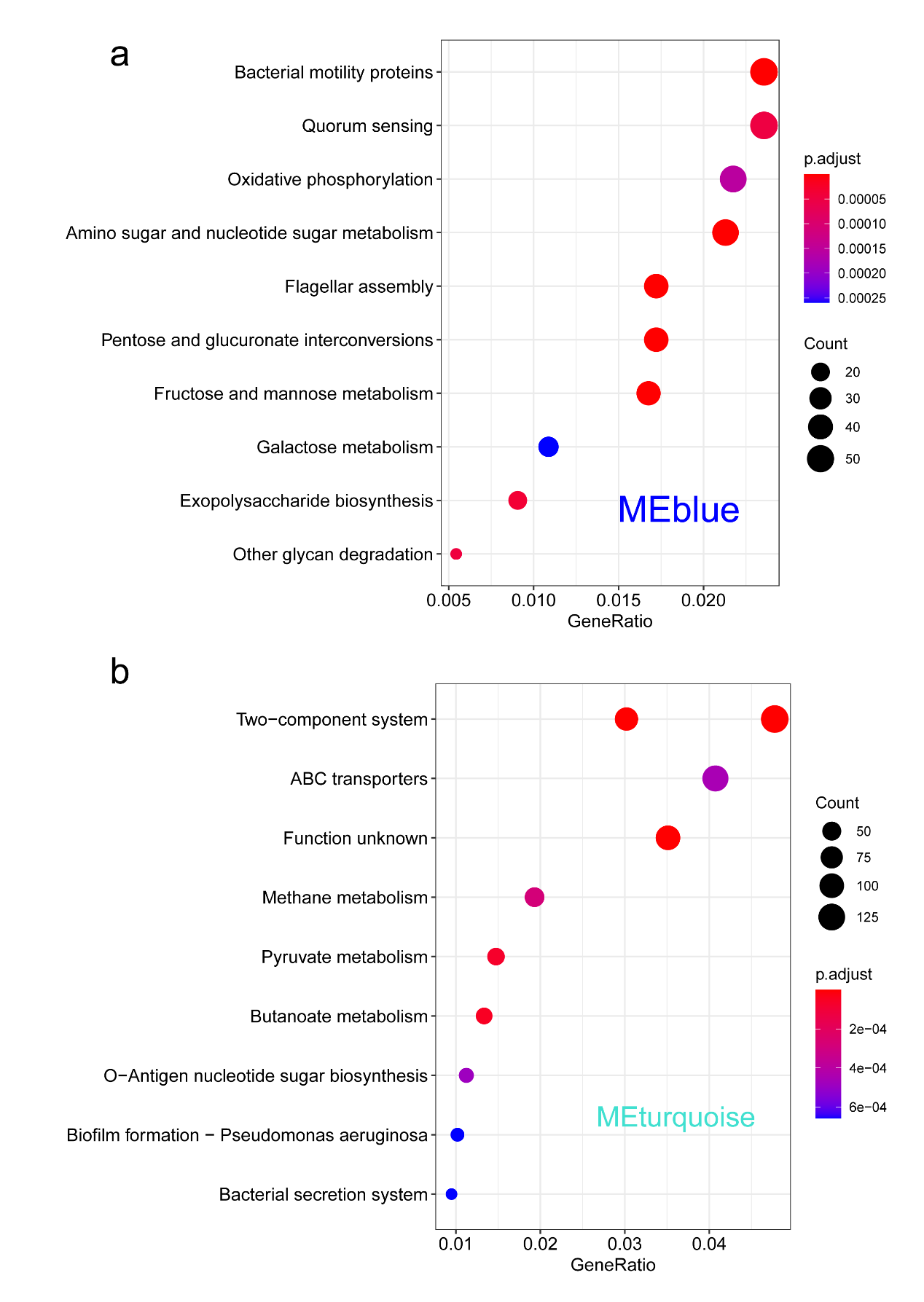


**Supplementary Figure S9.** **KEGG orthologs enrichment analyses**. a, Enriched pathways of MEblue that highly negative correlated with radiation. b, Enriched pathways of MEturquoise that highly positive correlated with radiation. Point size represented gene counts in the module and color represent significance. GeneRatio represented the proportion of enriched genes in total background genes within the same pathway.
